# On-chip integrated optical stretching and electrorotation enabling single-cell biophysical analysis

**DOI:** 10.1038/s41378-020-0162-2

**Published:** 2020-06-15

**Authors:** Liang Huang, Fei Liang, Yongxiang Feng, Peng Zhao, Wenhui Wang

**Affiliations:** 1 Department of Precision Instrument, State Key Laboratory of Precision Measurement Technology and Instrument, Tsinghua University, Beijing, China; 2 School of Instrument Science and Opto-Electronics Engineering, Hefei University of Technology, Hefei, China

**Keywords:** Engineering, Physics

## Abstract

Cells have different intrinsic markers such as mechanical and electrical properties, which may be used as specific characteristics. Here, we present a microfluidic chip configured with two opposing optical fibers and four 3D electrodes for multiphysical parameter measurement. The chip leverages optical fibers to capture and stretch a single cell and uses 3D electrodes to achieve rotation of the single cell. According to the stretching deformation and rotation spectrum, the mechanical and dielectric properties can be extracted. We provided proof of concept by testing five types of cells (HeLa, A549, HepaRG, MCF7 and MCF10A) and determined five biophysical parameters, namely, shear modulus, steady-state viscosity, and relaxation time from the stretching deformation and area-specific membrane capacitance and cytoplasm conductivity from the rotation spectra. We showed the potential of the chip in cancer research by observing subtle changes in the cellular properties of transforming growth factor beta 1 (TGF-β1)-induced epithelial–mesenchymal transition (EMT) A549 cells. The new chip provides a microfluidic platform capable of multiparameter characterization of single cells, which can play an important role in the field of single-cell research.

## Microfluidics: multiparameter cell characterization

A microfluidic chip utilizing optical fibers and electrodes can determine the mechanical and electrical parameters of different cell types. The biophysical behavior of cells are key markers for cellular identification, as well as for the study of diseased cells. Here, a team led by Wenhui Wang from Tsinghua University report a microfluidic chip that enables simultaneous probing of the mechanical and electrical behavior of single cells. Their device features two optical fibers to induce optical trapping and stretching of the cell, and four electrodes to measure electrical properties and for electrical rotation. They demonstrate the device for five different cell types, and study changes in the properties of cancerous cells.

## Introduction

Among many biophysical properties^1^, mechanical^2^ and electrical^3^ properties are intrinsic markers^4^ that can be used as cellular-specific characteristics in disease diagnostics^5^, drug screening^6^, and personalized medicine^7^. Continuing efforts have been made to measure the mechanical and electrical properties of cells and have resulted in many exciting technological advances^8,9^. Recently, as single-cell analysis has arisen, precise manipulation and analysis of single cells with the aid of microfluidics has become popular^10–12^. Typically, a microfluidic chip integrates multiple miniaturized modules to allow both manipulation and analysis of single-cell samples^13–15^. Basically, there are two ways to measure multiple biophysical parameters. One way is to serially connect the outlet of one device to the inlet of the next. This approach is straightforward, but the identity of cells must be tracked as they cross devices. Another way is to combine different measurement techniques into the same integrated space such that different properties can be measured simultaneously. This is the strategy that we follow in this paper.

The mechanical properties of cells influence cellular and subcellular functions, such as cell adhesion, migration, polarization and differentiation^16^, providing clues about cellular states and cell-related diseases^17^. Common cell mechanical properties include shear modulus, steady-state viscosity, and so on^18^. The main measurement methods such as atomic force microscopy^19^, magnetic twisting cytometry^20^, and particle-tracking microrheology^21^ function to probe forces at localized points of the cell. The major advantage of these methods is their capability to conduct high-resolution measurements due to the subcellular-scale positioning of the force probes. However, the measurement result is highly dependent on the probing position, representing the localized rather than whole-cell property.

Over the years, many microfluidic chips have been reported to measure the mechanical properties of a whole cell in suspension^22^. Basically, depending on how to apply forces on the cell, these chips can be based on mechanical, acoustic, and optical means. The mechanical means mainly include micropipette aspiration tests^23^ and cell transit analyzers^24^. Optical means mainly include optical tweezers^25^ and optical stretching^26^. Among mechanical means, micropipette aspiration tests use a micropipette to aspire the cell and measure the mechanical properties by analyzing its deformation. Suffering from low throughput, this approach is potentially harmful to cells and requires delicate operation. Cell transit analyzers measure the mechanical properties by measuring the time that the cell passes through a narrow microchannel. This approach has high throughput, but the microchannel is prone to blockage, and the size of cells is strictly limited.

Acoustic methods^27^ measure the mechanical properties of cells by calculating the time it takes for acoustic waves to travel within the cell. The advantage is noninvasive measurement and low acoustic intensity that does not interfere with cells. However, the method requires the piezo-transducer to be close to the adherent cells, which poses challenges for combination with microfluidic technology.

Among optical means, optical tweezers use focused laser beams to stretch cells by manipulating two small silica beads adhered to the opposite faces of a single cell to serve as handles for optical traps; cells are deformed by seizing the beads with twin optical tweezers and increasing the distance between the two focal spots. The advantage of this method is that cells are not directly exposed to the high optical intensity of the focal spots, which could cause the temperature to rise, thus minimizing optical damage to the cells. However, this technique requires sophisticated instruments, and the measurement throughput is low. Moreover, due to the size of microbeads, the measured results only reflect the local mechanical properties of cells. In contrast, optical stretching forms an optical trap based on two opposing laser beams with a Gaussian intensity distribution, capable of capturing the cell in suspension and stretching it in situ^28,29^. Because the laser beam in optical stretching is divergent and unfocused, the radiation damage to the cell of interest is minimal. Furthermore, optical stretching acts on the entire cell membrane, so the whole-cell measurement of viscoelastic properties is enabled. Overall, optical stretching is well established and widely used.

In addition to mechanical properties, cellular electrical properties are often used to describe the viability, growth, and identity of different cell types^30^. Electrical parameters are closely related to the structure and chemical composition of cells and can be used to study their physiology. Cellular electrical parameters mainly include the conductivity and permittivity of the cell membrane and cytoplasm^31^. Currently, the electrical properties of cells can be achieved by a variety of methods, including electrical cell-substrate impedance sensing^32^, impedance flow cytometry^33^, and electrorotation methods^34–36^, where electrorotation is the only method for accurately extracting the electrical properties of cell membranes and internal cytoplasm.

Accordingly, we aim to integrate the optical stretching modality on top of electrorotation platforms. Electrorotation can be implemented by planar electrodes or 3D electrodes. In particular, 3D electrodes generate a much more uniform electric field distribution in the vertical direction^37,38^ than planar electrodes. Thus, the 3D electrode configuration would result in a consistent and stable rotation speed for the cell, avoiding concerns about the cell’s position in the vertical direction. Extended from the basic idea reported in microTAS 2018^39^, this paper presents an integrated microfluidic chip that embeds two extra opposing optical fibers between four isolated 3D electrodes. The two optical fibers are used to fulfill both optical trapping and stretching for mechanical property measurement, while the four 3D electrodes are deployed for electrorotation and dielectric property measurement. Meanwhile, the 3D electrode structure here facilitates insertion and alignment of the optical fibers. Enabled by the unique structure, the device thus offers the advantages of (i) multiple manipulation of capture, then trap–stretch, and then trap–rotate; and (ii) separate in situ measurement of mechanical and electrical properties for the same cell remaining in the device. The mechanical and electrical properties of the same single cell are thus obtained and can be linked for single-cell characterization.

In the sequel, we first elaborate the working principle in line with the device design. Then, we demonstrate single-cell capture under the effect of optical forces and show the cellular stretching step-stress responses of different types of cells under different optical powers. In this study, we tested five cell types (HeLa, A549, HepaRG, MCF7, and MCF10A) available to us and biologically representative (cancer cells, normal cells; human cells, and animal cells) to show the feasibility and potential impact of our method for a wide range of cells. According to the stretching deformation results, we could calculate the mechanical parameters of the cells by fitting the step-stress response curves. Based on the stable trap but no stretching of cells, we obtained the rotation spectrum of the cells and extracted their dielectric properties (the area-specific membrane capacitance and the conductivity of cytoplasm). We also showed that the mechanical and electrical properties could be linked to characterize cells. To show the great potential of our platform, we further measured the mechanical and electrical properties of A549 cells treated with TGF-β1, which is believed to induce epithelial–mesenchymal transition (EMT) in cancer development and progression^40^. The measurement results confirm that EMT A549 cells change their mechanical characteristics and, more importantly, provide evidence that EMT induces changes in cellular electrical properties for the first time. The unprecedented manipulation and measurement capabilities lend this enhanced platform great potential to investigate single cells from new angles.

## Materials and methods

### Concept design and working mode of the chip

**Fig. 1:**
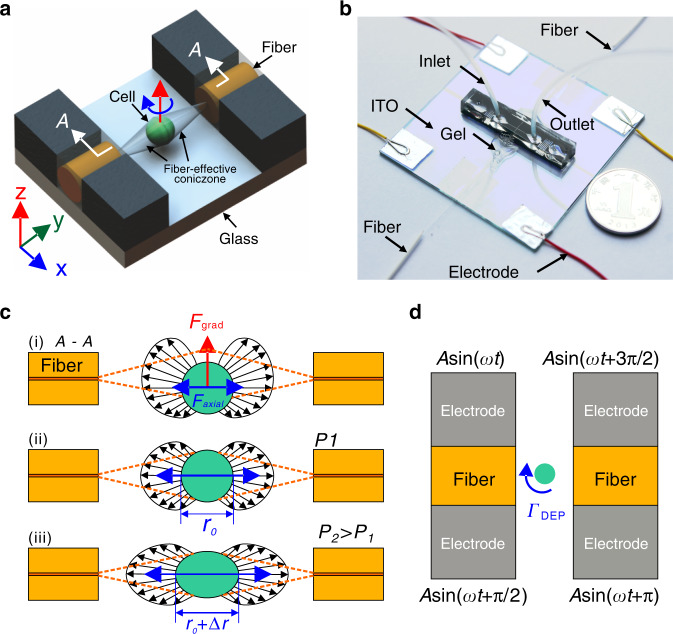
Chip design and working principle. **a** Sketch of the design, not to scale. **b** Photograph of the chip. **c** Working principle of optical trap and stretch. **d** Working principle of electrorotation

Figure 1a shows a sketch of the microfluidic chip. The four isolated 3D sidewall electrodes sitting above an indium tin oxide (ITO)-patterned glass slide are used to generate an in-plane rotating electric field, in which a virtual cell rotating chamber is formed. The four electrodes also stand as microchannel walls, forming two orthogonal microchannels. The microchannel along the *X*-axis is the main channel through which the cell sample flows. The perpendicular channel along the *Y*-axis is the optical fiber loading channel, which embeds two *Y*-axis aligned single-mode fibers for cell trapping and stretching. Figure 1b shows a photograph of the microfluidic chip. The microchannels are walled by carbon-PDMS (C-PDMS) electrodes. Two optical fibers are inserted into the fiber loading microchannel and aligned well.

The chip is operated in a sequential capture–trap–stretch–relax–rotate–release mode (Fig. 1c), but the stretching is not performed during rotation of the cell. Once flowing into the fiber-effective zone along the main channel, the cell is captured and trapped by the optical fiber laser-induced optical force to an equilibrium position, which is presumably the intersection point of the two orthogonal channels, or the center of the rotation chamber. Upon trapping, the fluid is stopped. Then, by applying a high-power laser, the cell will be stretched in situ along the optical fiber direction (*Y*-axis). Its time-varying deformation can be instantly recorded by a camera, from which its mechanical properties can be extracted. After stretching is performed, the cell is given sufficient time to restore its morphology by resetting the laser power low enough to maintain the trapping status. Next, AC signals configured with proper parameters are applied to the four 3D electrodes to rotate the cell in-plane (Fig. 1d). Again, the camera is used to obtain the rotation spectrum, namely, the cellular rotation speed in relation to the AC signal parameters, from which the electric properties of the cell can be extracted. After the above procedure for a specific cell sample, the laser and AC signals are turned off, and the fluid starts flowing again to release the current cell and bring up the next one.

### Working principle of optical stretching and mechanical property measurement

Optical stretching is performed through two counterpropagating fiber laser beams, which are combined to trap and deform the cells using optically induced forces^41^. As shown in Fig. 1c, there are two orthogonal induced forces, gradient force and axial force. The gradient force pulls the cell toward the position where the laser intensity is the greatest, i.e., the centerline of the optical fibers (Fig. 1c-i). The axial forces push the cell away from both optical fiber ends until the cell is trapped at an equilibrium point along the optical fiber centerline, like tug-of-war (Fig. 1c-ii). When the laser power is increased, the forces acting on the cell will correspondingly increase such that the cell becomes stretched in the direction of light propagation (Fig. 1c-iii). Tracking the cell contour enables dynamic measurement of the cell deformation during the stretching process, from which the cell mechanical properties such as shear modulus can be extracted. An effective measurement method to characterize the mechanical properties of a cell is through step-stress analysis^42,43^. In step-stress analysis, the cell is initially trapped at the equilibrium position by optical stretching at a low power condition. Subsequently, the cell is subject to a higher laser power held for a constant time to deform the cell in its elasticity range, and the corresponding deformation is recorded. The laser power is finally restored to the original low power to keep the cell trapped and allow its relaxation to the original morphology.

Denoting by ***γ***(*t*) the strain of the cell as a function of time, it is given by ${\boldsymbol{\gamma}} \left( {\boldsymbol{t}} \right) = \frac{{{\boldsymbol{r}}\left( {\boldsymbol{t}}\right) - {\boldsymbol{r}}_{\mathbf{0}}}}{{\boldsymbol{r}}_{\mathbf{0}}}$, where *r*(*t*) is the time-varying diameter of the cell along the beam axis, and *r*_0_ is the original diameter of the cell along the same direction when the cell is trapped initially. These values are measured through a camera imaging system.

In the viscoelastic model, the strain of the cell is related to its viscoelastic properties by the following equation^44^:

1 $${\boldsymbol{\gamma}} \left( {\boldsymbol{t}} \right) = \sigma_{0}\left( {\frac{{b_{1}}}{{a_{1}}} - \frac{{a_{2}}}{{a_{1}^{2}}}} \right)\left( {{\mathbf{1}} - {\mathbf{exp}}\left( -{\frac{a_{1}}{{a_{2}}}{\boldsymbol{t}}} \right)} \right) + \frac{\sigma_{0}}{a_{1}}{\boldsymbol{t}}$$

where *a*_1_, *a*_2_, and *b*_1_ are the coefficients to be solved. *σ*_*0*_ is the maximum stress applied to the cell along the beam. It can be calculated by

2 $${\boldsymbol{\sigma_{0}}} = \frac{{n}_{med}I_0}{c}\left( {{\mathbf{2}} - {\boldsymbol{R}} + {\boldsymbol{R}}^2} \right)\left( {\frac{n_{cell}}{n_{med}}- 1} \right)$$

where *c* is the speed of light in vacuum (*c* ≈ 2.99 × 10^8^ m/s), *n*_med_ is the refractive index of the medium (typically *n*_med_ ≈ 1.335), and *n*_cell_ is the refractive index of the cell. *R* is the amount of light reflected at the interface between the medium and the cell, and it can be calculated by

3 $$R = \left( {\frac{{n_{\mathrm{cell}} - n_{\mathrm{med}}}}{{n_{\mathrm{cell}}} + n_{\mathrm{med}}}} \right)^2$$

Finally, *I*_0_ is the laser intensity along the laser axis at the position of the cell and can be calculated using the following equation:

4 $${\boldsymbol{I}}_0 = \frac{{2{\boldsymbol{P}}}}{{\omega ^2\pi }}$$

where *P* is the total power of the laser beams, and *ω* is the radius of the laser beams at the position of the cell.

Upon laser power retraction, the cell relaxes dynamically back to equilibrium status, and the strain can be fitted by

5 $${\boldsymbol{\gamma}} \left( {\boldsymbol{t}} \right) = {{\boldsymbol{\sigma}}_{0}}\left( {\frac{{\boldsymbol{b}}_{1}}{{\boldsymbol{a}}_{1}} - \frac{{\boldsymbol{a}}_{2}}{{\boldsymbol{a}}_{1}^{2}}} \right)\left( 1 - {{\mathbf{exp}}\left(-{\frac{{{\boldsymbol{a}}_1}}{{{\boldsymbol{a}}_2}}{\boldsymbol{t}}} \right)} \right){\mathbf{exp}}\left( - {\frac{{\boldsymbol{a}}_{1}}{{\boldsymbol{a}}_2}\left( {{\boldsymbol{t}} - {\boldsymbol{t}}_1} \right)} \right) + \frac{{\boldsymbol{\sigma}}_{0}}{{{\boldsymbol{a}}_1}}{\boldsymbol{t}}$$

Fitting the experimental strain data with the model described by Eqs. (1) and (5), the coefficients *a*_1_, *a*_2_, and *b*_1_ can be determined, from which typical rheological parameters such as shear modulus *G*, steady-state viscosity *ψ*, and relaxation time *τ* are given by

6 $$G = \frac{1}{{2\left( {1 + \mu } \right)}}\frac{{a_1^2}}{{a_1b_1 - a_2}}$$

7 $$\psi = \frac{1}{{2\left( {1 + \mu } \right)}}a_1$$

8 $${\boldsymbol{\tau}} = {\boldsymbol{b}}_1$$

where *μ* is Poisson’s ratio, which may be assumed to be *μ* = 0.45–0.50 for biological material. In our experiment, we set *μ* = 0.50. The three mechanical parameters can be collectively used to explain the different deformability among cells. In particular, the shear modulus and steady-state viscosity reflect the ability of the shell-like actin cortex as the main module resisting small deformations. The relaxation time can be related to the transient binding of actin crosslinking proteins. Though not independent, the three mechanical parameters are more detailed indicators for deformability^44^.

### Working principle of electrorotation and dielectric property measurement

According to DEP theory, by applying AC signals with 90° phase shifts to the four vertical electrodes, an in-plane rotating electric field is formed in the virtual rotation chamber enclosed by the electrodes. The cell inside this chamber will exhibit in-plane rotation corresponding to the rotating electric field. The cell rotation speed is related to the electrical parameters of the cell, solution, and external electric signals^45^, given by

9 $$\Omega = \frac{{\varepsilon _m}}{{2\eta }}{\mathrm{Im}}\left[ K_{{\mathrm{CM}}} \right] \cdot E^2$$

where *η* is the viscosity of the medium, *ε*_*m*_ is the permittivity of the solution, and

10 $$K_{{\mathrm{CM}}} = \frac{{\varepsilon _{\boldsymbol{c}}^ \ast - \varepsilon _{{m}}^ \ast }}{{\varepsilon _{\boldsymbol{c}}^ \ast + 2\varepsilon _{{m}}^ \ast }}$$

11 $$\varepsilon _m^ \ast = \varepsilon _m - j\frac{{\sigma _m}}{\omega }$$

12 $$\varepsilon _c^ \ast = \varepsilon _c - j\frac{{\sigma _c}}{\omega }$$

where *K*_CM_ is the Clausius–Mossotti (CM) coefficient, *σ*_*m*_ is the conductivity of the solution, *ε*_*c*_ is the permittivity of the cell, and *σ*_*c*_ is the conductivity of the cell.

The cell is mainly composed of the cell membrane and cytoplasm, and its electrical properties are also more complex models. It is assumed that the cytoplasm has a uniform internal structure and can be equivalent to a single-shell model.

13 $$\varepsilon _c^ \ast = \varepsilon _{\mathrm{mem}}^ \ast \frac{{\left( {\frac{R}{{R - d}}} \right)^3 + 2\left( {\frac{{\varepsilon _{cyto}^ \ast - \varepsilon _{\mathrm{mem}}^ \ast }}{{\varepsilon _{\mathrm{cyto}}^ \ast + 2\varepsilon _{\mathrm{mem}}^ \ast }}} \right)}}{{\left( {\frac{R}{{R - d}}} \right)^3 - \left( {\frac{{\varepsilon _{\mathrm{cyto}}^ \ast - \varepsilon _{\mathrm{mem}}^ \ast }}{{\varepsilon _{\mathrm{cyto}}^ \ast + 2\varepsilon _{\mathrm{mem}}^ \ast }}} \right)}} \approx C_{\mathrm{mem}}^ \ast \frac{{R \cdot \varepsilon _{\mathrm{cyto}}^ \ast }}{{R \cdot C_{\mathrm{mem}}^ \ast + \varepsilon _{\mathrm{cyto}}^ \ast }},\left( {d\ll R} \right)$$

where $\varepsilon _{\mathrm{mem}}^ \ast = \varepsilon _{\mathrm{mem}} - j\frac{{\sigma _{\mathrm{mem}}}}{\omega }$, $\varepsilon _{\mathrm{cyto}}^ \ast = \varepsilon _{\mathrm{cyto}} - j\frac{{\sigma _{\mathrm{cyto}}}}{\omega }$, *ε*_mem_ is the cell membrane permittivity, *σ*_mem_ is the cell membrane conductivity, *ε*_cyto_ is the cell cytoplasm permittivity, *σ*_cyto_ is the cell cytoplasm conductivity, and $C_{\mathrm{mem}}^ \ast = C_{\mathrm{mem}} - j\frac{{G_{\mathrm{mem}}}}{\omega }$. $C_{\mathrm{mem}} = \frac{{\varepsilon _{\mathrm{mem}}}}{d}$ is the area-specific membrane capacitance, where $G_{\mathrm{mem}} = \frac{{\sigma _{\mathrm{mem}}}}{d}$ is the area-specific membrane conductance, and *d* denotes the membrane thickness. Area-specific membrane capacitance reflects the membrane composition and morphological complexity of the cell and can be related to the accumulated ionic charges on the cell membrane in response to the applied electrical field. The conductivity of the cytoplasm reflects the electrical mobility of ionic species in cytoplasmic water and intracellular barriers^32^.

The electrical properties of the cell can be calculated by fitting the experimental data with the theoretical model^46^. According to the electrorotation spectra measured in the electrorotation experiment, the parameter fitting method is used to minimize the residual error by

14 $$\min {\mathop{\sum}\limits_{i}} \left[{\Omega} _{\mathrm{exp}}\left( \omega _{i} \right) - \Omega _{\mathrm{theory}}\left( \omega _{i} \right) \right]^{2}$$

It is worth emphasizing that the 3D electrodes used in the chip can overcome the attenuation problem^36^ of the electric field in the vertical direction of the planar electrode, so the cell can have a more uniform rotation speed at different heights. Hence, the cell rotation spectrum measured will be more accurate than that of planar electrodes.

### Experimental setup

Two counterpropagating laser beams emerging from single-mode optical fibers are combined to trap and deform cells using optically induced forces. The primary laser produced by a 980-nm pump laser (VLSS-980-B, Connet Fiber Optics, China) is split into two single-mode optical fibers (HI1060, Thorlabs) by a 1 × 2 (50:50) coupler, which are then aligned along the microchannel. The choice of laser wavelength is crucial to avoid a temperature increase in the medium due to light absorption by water. The laser wavelength of 980 nm has a low absorption coefficient such that the heat problem can be ignored^47^. To prevent the pump laser from being burned by the returning laser, an isolator (OI-980, Connet Fiber Optics, China) is connected between the output port of the pump laser and the coupler. The 3D electrodes are connected to a four-channel signal generator (TGA12104, TTi, England). The cell solution is driven by gravity, which is implemented by adjusting the relative height of a reservoir by a manipulator (Sutter MP-285, USA). The chip is placed under an inverted microscope (Ti-U, Nikon), whose C-mount side port is connected to a CCD camera (acA640-120gm, Basler, Germany) working at 30 Hz for experimental process observation and recording. To prevent laser glaring when the cell is situated under the laser beam, a 980-nm filter is placed between the chip and CCD camera. The whole setup sits on a vibration-isolation optical table.

### Chip fabrication

3D electrodes were fabricated with C-PDMS^36,38^, which was obtained by mixing carbon black particles into a PDMS mixture (10:1 (by weight) PDMS base/curing agent) (Sylgard 184, Dow Corning) and mixing for 15 min by hand until a homogeneous paste was obtained^48^. A 125-μm-thick layer of negative photoresist (SU8-2075, MicroChem) was first spin-coated and photopatterned to obtain a channel mold. C-PDMS was then plastered on the mold with a blade to remove the excess conductive polymer. After being cured (80 ^°^C, 30 min), pure PDMS was poured on the mold and cured again. Finally, the PDMS/C-PDMS channel was unmolded and bonded to the ITO glass substrate with patterned electrodes. The patterned electrodes on ITO glass were fabricated by photolithography with negative photoresist (BN303-30, Kempur) and wet etching to ensure electric contact with the C-PDMS electrodes and form external-wiring pads. Oxygen plasma was then used to enhance the adhesion of ITO glass and C-PDMS. The width and height of the optical fiber loading channel were designed to be 125 × 125 μm. Constrained by the microchannel geometry, two optical fibers of 125 μm in diameter were inserted with good alignment after bonding of the ITO glass and C-PDMS. To prevent possible leakage through the gap between the round optical fiber and the square channel, we sealed the outside of the channel with adhesive.

The adhesive (UHU, Germany) was simply plastered on both entrances of the two fiber loading channels. Due to its high viscosity, the adhesive would not run into the main channel while filling the gap. The two optical fibers were carefully aligned by tweaking around the fiber outside of the channel. The movement of the fibers was done by carefully touching the fiber with a steel needle with a 40-nm-positioning-resolution micromanipulator (Sutter MP-285). Note that the movement was operated immediately after the adhesive deposition, when the adhesive was still liquid and soft enough for the fibers to be moved. Once the alignment was done, the two fibers were temporarily fixed to the glass by adhesive tape, which may be finally removed. The device was then allowed to sit still at room temperature for 0.5 h to allow solidification of the adhesive such that the fibers were fixed firmly in position. According to our experiment (ten times), the tape kept the alignment of the fibers nearly the same while the adhesive was solidifying. Therefore, this alignment procedure is highly repeatable. The *y*-axis alignment was confirmed by direct microscopic observation, while the *z*-axis alignment was confirmed by measuring the optical coupling power, which is a standard method of optical fiber alignment. We applied a certain power laser to one optical fiber and measured the output power from the other optical fiber. The two optical fibers are said to be aligned when the input power equals the output power. This method was validated by sacrificing one fabricated device and looking at the cross-section of the two aligned optical fibers. The misalignment offset was controlled below 2 μm after the operator practiced to become skillful.

### DEP buffer preparation

Different media have different viscosities, absorbances, and refractive indices, which have certain effects on optical stretching and rotation. Therefore, to ensure the consistency of the measuring conditions and comparability of the measurement results, the same solution was always used in the experiment. The buffer solution should have a low conductivity, which can reduce the DEP-induced Joule heating effect. In this work, DEP buffer was composed of 16% (w/v) sucrose + 2% (w/v) PBS + 82% (w/v) deionized water, which had a conductivity of 36.5 mS/m and was used to dilute the cell suspension.

### Cell preparation

HeLa, A549, HepaRG, MCF7, and MCF10A cells 8–17 μm in diameter were used in the experiment. These cells were cultured in a 5% CO_2_ and 37 °C incubator. Cells were rinsed with PBS twice and lifted off by treatment in trypsin for 5 min. Then, the cell suspension was washed three times by centrifuging at 300 × *g* for 5 min, removing the supernatant with a pipette, and resuspending the cell pellet in 1 ml DEP buffer. To increase the efficiency of single cell capture and reduce the frequency of trapping multiple cells, we diluted the cell concentration to 1.5 × 10^5^ cells/ml.

### TGF-β1 treatment

A549 cells were selected for the EMT experiment. Cells were cultured in DMEM (Gibco). Each medium was supplemented with 10% FBS (Gibco) and 100 U/ml penicillin–streptomycin (Gibco). To induce EMT, cells were treated with 5 ng/ml recombinant human TGF-β1 (Peprotech, USA) for 48 h according to the product user guide.

## Results and discussion

### Cell capture–trap–stretch by optical stretching

The flowing cell in the medium could be captured, trapped, and stretched smoothly by optical fibers. As predicted by the theory, when one cell entered the effective zone of the two optical fibers, collective action of both gradient and axial forces drove the cell to the center of the chamber.

**Fig. 2:**
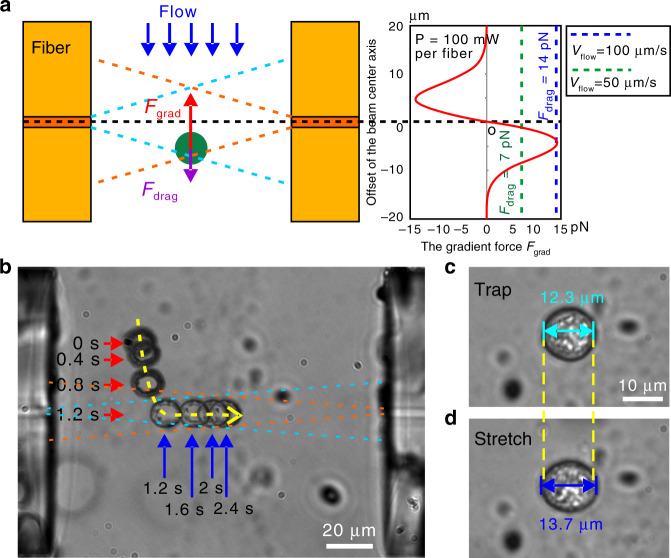
**Cell trapping and stretching**. **a** Schematic diagram of laser propagation in optical stretching and the distribution of the gradient force along the center of the channel. **b** Two-beam laser traps one MCF7 cell from the side into the center of the main microchannel under the collective action of gradient and axial forces. **c** Cell being trapped at a lower power (100 mW per fiber). **d** Cell stretching at a higher power (400 mW per fiber)

The key factor for the cell to be captured lies in the flow rate, which should be limited to avoid the flow drag force being greater than the gradient force produced by the optical fibers^49^. When flowing along the channel centerline, the cell is subject to the maximum drag force as the flow velocity across the channel cross-section profile is parabolic, as shown in Fig. 2a. In this paper, defining the conic zone as the overlapping region of the two laser propagation areas, for a cell of *r* in diameter, its gradient force at the center point of the conic zone is estimated to be 14 pN (*P* = 100 mW per fiber) according to the literature^50^. The equivalent drag force would result in a maximum flow rate of 100 μm/s. Figure 2a shows the distribution of the gradient force along the center of the channel. Using this value as a starting point, we adjusted the flow rate through experiment and found that 40 μm/s worked well for all five types of cells. For the captured cell to be trapped in the center, the laser power should be selected to produce enough gradient force to counter against the sum of gravity and buoyancy force of the cell. When approaching the conic zone from the side of the channel, the cell can also be captured. Normally, the cell could be trapped at the center within 3 s upon capture by the conic zone under the current chip structure.

In the experiment, only those cells dispersed in the height overlapping with the conic zone could be trapped. These cells were a small portion according to the probability principle; thus, the trapping percentage was low. However, there may be a high cell-trapping percentage in some scenarios. To this end, many efficient particle focusing methods^51–53^ could be integrated into the device to bring cells in the vicinity of the conic zone. Furthermore, cell dimension has little effect on the trapping percentage, as long as the cell is smaller than the conic zone, which was the case for this work. The reason is because the cell trap is the result of balancing the fluidic force and the light gradient force, and both forces are proportional to the cell dimension^47,54^. Therefore, under the used experimental settings (i.e., laser power of 200 mW and flow rate of 40 μm/s), the five types of cells flowing into the conic zone of the optical fibers were almost 100% trapped.

If the two optical fibers are not aligned well, there will be several interesting motion patterns in line with the misalignment situations. We skip this part here because it is not the focus.

Figure 2b shows the capture–trap process of an MCF7 cell (ESI Video 1). Note the effect of using the filter for glare removal. First, the cell approached the effective conic zone of the two optical fibers along the fluidic flow. Once entering the conic zone, the cell was dragged with acceleration to the centerline of the aligned optical fibers under the gradient force, and then, the cell was pushed to the center of the main channel by the scattering axial force. For the case where the two optical fiber ends were aligned well with the channel walls, the cell was centered adaptively and trapped steadily in the main channel. After the cell was trapped (Fig. 2c), increasing the laser power stretched the cell along the fiber centerline (Fig. 2d).

### Mechanical property measurement via optical stretching

**Fig. 3:**
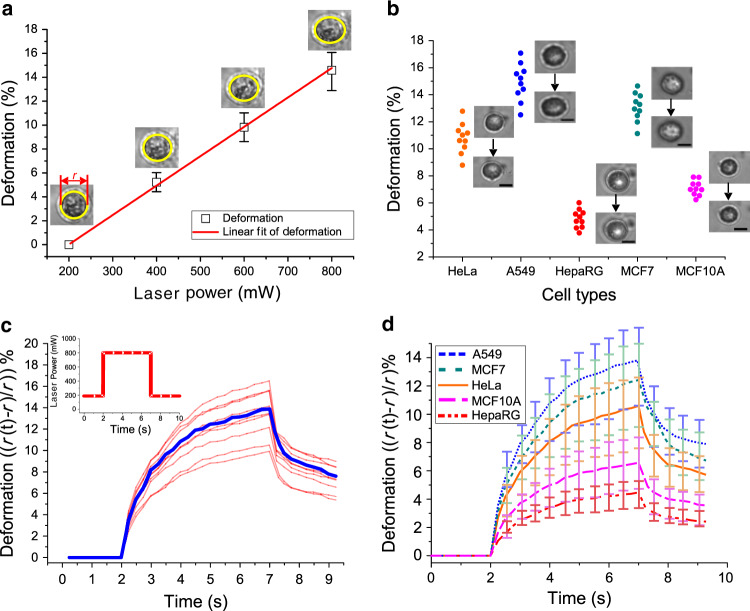
**Optical stretching-induced cell deformation and step-stress responses of cells under optical stretching**. **a** The maximum strain vs. laser power for the A549 cell, which was subjected to step-stress experiments with 200, 400, 600, and 800 mW laser power at a constant time of 20 s. **b** The maximum deformation of ten samples for the five types of cells with 800 mW laser power at a constant time of 10 s. Cell morphology before/after trap–stretch is shown for each cell type. Scale bar: 10 μm. **c** The step-stress response curves of ten A549 cell samples. The blue line outlines the average response of the ten cells. **d** The average step-stress responses of five different types of cells. Error bars in **a** and **d** correspond to the standard deviation for ten cell samples

The strain of the cell was measured offline through the video clips recorded in synchronization with the step-stress deformation experiment. As mentioned before, once trapped by the fiber lasers with a low power (e.g., 200 mW in total), the cell was stretched along the direction of light propagation by setting a higher laser power (e.g., up to 800 mW in total) for a constant time. During the course of stretching, the images of the cell were recorded and analyzed offline by commonly used image software (ImageJ, NIH) to determine the strain. We observed the dynamic stretching pattern for the cell, which was subjected to several cycles of step-stress experiments. In each cycle, the cell subjected to the low laser power for trapping was stretched by increased laser power for the same constant time (i.e., 20 s) and then returned to the original low power for 1 min to allow cell relaxation. The maximum strain occurred at the end of the constant time. Figure 3a shows the maximum stretching deformation of ten A549 cells at different power levels of 200, 400, 600, and 800 mW. It was found that the maximum strain linearly correlated with the applied laser power, which is consistent with the literature^55^. Specifically, the maximum deformation at 800 mW was ~3 times that at 400 mW. This observation was critical because it indicates that we do not have to worry about the stretching effect variance across laser powers. Instead, as long as the cell stays viable, we can set the laser power as high as possible to induce paramount cellular deformation, which facilitates the measurement accuracy.

In this work, we chose a sample size of 10 to demonstrate the repeatability of the mechanical and electrical property measurements. The sample size was comparable to the size in the literature^18,56^ for comparison. We obtained the maximum strain for the five types of cells to reflect their deformability (ESI Video 2). For each type, we tested ten samples with a laser power of 800 mW at a constant time of 10 s. Under such conditions, the cells were observed to recover their normal morphology upon relaxation after stretching, and their viability reached 90% (compared with 95% for the control) via the Trypan blue exclusion assay. Figure 3b shows the maximum deformation of the five types of cells. We found that the deformability of the HeLa, A549, and MCF7 cells (cancer cells) was significantly larger than that of the HepaRG and MCF10A cells (normal cells). The average deformation of A549 was the largest, ~14.7%, and that of HepaRG was the smallest, ~4.8%. Interestingly, the average deformation of MCF7 cancer cells is ~2 times that of MCF10A normal cells.

We then measured the time-varying strain curves of the five types of cells to extract their mechanical properties by a step-stress experiment. For each type, we tested ten samples with a laser power of 800 mW for a step constant time of 5 s. Thus, we obtained ten step-stress curves and the average curve for each cell sample (ESI Video 3). Figure 3c shows the sample-wise and type-wise step-stress responses of A549 cells, and Fig. 3d shows the type-wise step-stress responses of all five types of cells. The deformation of cancer cells (A549, MCF7, and HeLa) is greater than that of noncancer cells (MCF10A and HepaRG), indicating that cancer cells are more deformable than normal cells.

We obtained three mechanical property indicators for the five types of cells, as summarized in Table 1. The fitting errors in the table indicate a good match between the experimental data and the mathematical model. Using numerical methods, we obtained the sensitivity for the shear modulus (∼0.02 Pa), the sensitivity for the steady-state viscosity (∼0.03 Pa·s), and the sensitivity for the relaxation time (~0.03 s). In the literature^16^, MCF7 cells tested by optical stretching have an 18 Pa shear modulus, which is on the same order of magnitude as the result (16 Pa) in this study. Differences in the cell line culture environment and germline variance across different laboratories are always considered the factors for variance in measurement results. Therefore, we believe that the comparable results w.r.t. the literature can validate our measurement method.

**Table 1:** Mechanical property measurement results for five types of cells

Cell type	Shear modulus G (Pa)	Steady-state viscosity *ψ* (Pa ·s)	Relaxation time *τ* (s)
HeLa	19.10 ± 0.81	62.13 ± 4.30	3.50 ± 0.38
HepaRG	44.99 ± 1.97	156.37 ± 11.27	3.72 ± 0.42
MCF7	15.98 ± 0.69	52.07 ± 3.67	3.51 ± 0.39
MCF10A	30.67 ± 1.30	100.47 ± 6.83	3.53 ± 0.38
A549	14.63 ± 0.65	47.93 ± 3.37	3.53 ± 0.39

The results indicate that HeLa, A549, and MCF7 cancer cells have a lower shear modulus than HepaRG and MCF10A normal cells, further confirming that the cancer cells are softer. HepaRG cells have the highest steady-state viscosity and relaxation time. Therefore, the response times of HeLa deformation are the slowest among the five types of cells under investigation. In particular, comparing MCF10A cells with MCF7 cells, the shear modulus and viscosity of MCF10 cells are approximately twice those of MCF7 cells, and the relaxation time of MCF10A cells is slightly larger than that of MCF7 cells.

### Cell electrorotation and dielectric property measurement

**Fig. 4:**
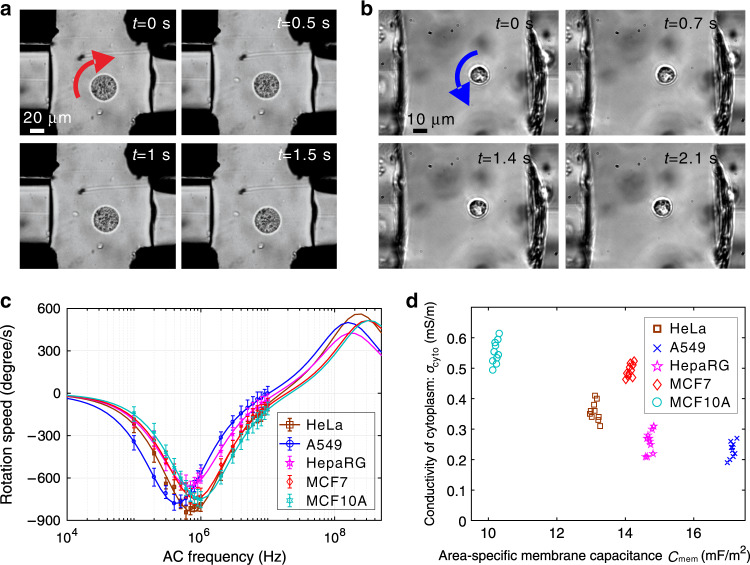
**Electrorotation-based cell dielectric property measurement of the five types of cell**s. **a** One HeLa cell rotating clockwise at ~120 degrees/s. **b** One HepaRG cell rotating anticlockwise ~45 degrees/s. Both cases used V*p* − *p* = 6 V and *f* = 600 kHz. **c** Cell rotation spectra (V*p* − *p* = 10 V). Each cell type had 10 samples. **d** The 2D clustering visualization of the two measured dielectric parameters

In-plane rotation of the five types of cells was implemented and controlled by adopting proper configuration of AC signals applied to the four 3D electrodes. To prevent the optical traps from affecting the cell rotation measurements, the laser needs to be switched off when the cell is rotating. In the experiment, after testing the rotation speed of one frequency point, we turned on the laser to recapture the cell and pull it back to the original capture position. By resetting its rotation position, the cell rotation spectrum was highly accurate and repeatable. In particular, we applied AC signals of ≤10 V and a frequency in the range of 100 kHz to 10 MHz for in-plane cell rotation experiments. We could rotate the cell at the trap site in two directions, i.e., clockwise and anticlockwise (ESI Video 4). Figure 4a shows one HeLa cell rotating clockwise at a speed of ~120°/s, and Fig. 4b shows one HepaRG cell rotating anticlockwise at a speed of 45°/s. We could also enhance the speed by increasing the voltage amplitude. However, as expected, the rotation spectrum is not positively related to AC frequency (ESI Video 5), which is swept in the dielectric property measurement.

For each type of cell, we tested ten samples under the AC signal V*p* − *p* = 10 V and frequency *f* = 100 kHz–10 MHz to obtain each type’s rotation spectrum, which had 20 data points (Fig. 4c). The rotation spectrum was then fitted by following the rule described by Eq. (14), which minimizes the fitting error. Finally, the fitting result is shown as the solid line in Fig. 4c, and the corresponding values for the two dielectric properties, the area-specific membrane capacitance *C*_mem_ and the cytoplasmic conductivity *σ*_cyto_, are shown in Fig. 4d. Table 2 also summarizes the measurement results of the dielectric parameters for the five types of cells. Consistent with previous measurements, there exists a significant difference in the dielectric properties of different types of cells. A549 cells have the largest area-specific membrane capacitance and the smallest conductivity of the cytoplasm. In contrast, MCF10A cells have the smallest area-specific membrane capacitance and the largest conductivity of the cytoplasm. As a comparison of the same lineage of cell lines, the average area-specific membrane capacitance of MCF7 is nearly 1.5 times that of MCF10A, but the conductivity of the cytoplasm of MCF7 is smaller than that of MCF10A. The possible reason is that cancer cells have rougher surfaces (wrinkles, frills, and grooves), which means that the total cell membrane area is significantly larger than that of normal cells^56^.

**Table 2:** Electrical property measurement results for five types of cells

Cell type	Area-specific membrane capacitance *C*_mem_ (mF/m^2^)	Conductivity of cytoplasm *σ*_cyto_ (S/m)
HeLa	13.23 ± 0.32	0.35 ± 0.06
HepaRG	14.71 ± 0.15	0.23 ± 0.07
MCF7	14.15 ± 0.09	0.50 ± 0.03
MCF10A	10.16 ± 0.07	0.55 ± 0.02
A549	17.12 ± 0.14	0.23 ± 0.04

### Multiparameter measurement result analysis

**Fig. 5:**
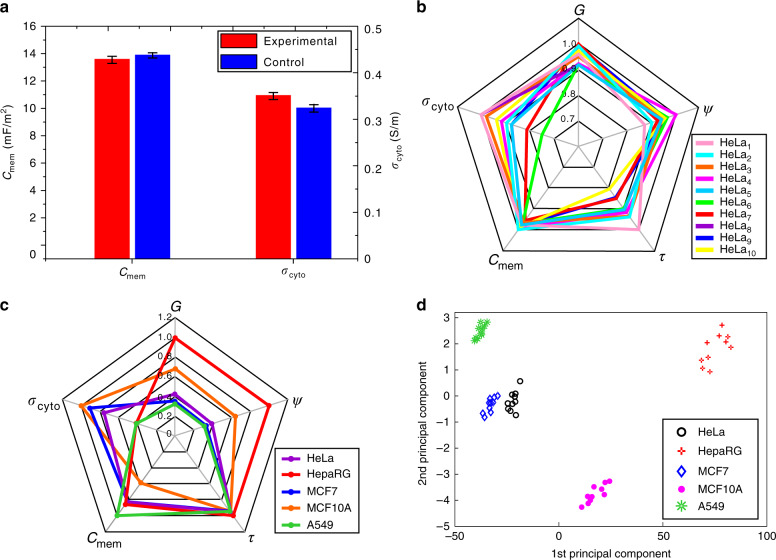
**Multiparameter measurement result analysis**. **a** Comparison of the electrical property measurement results of ten HeLa cell samples between the experimental and control groups. **b** The distribution of all five mechanical and electrical parameters of ten HeLa cell samples. **c** The distribution of all five mechanical and electrical parameters of the five types of cells. **d** PCA results of the five parameters of the five types of cells

To evaluate the effect of stretching on the electrical property measurement, we performed a control experiment with HeLa cells. In the control experiment, ten HeLa cells were solely electrorotated without stretching for direct electrical property measurement. The control measurement results were *C*_mem_ = 13.61 ± 0.16 mF/m^2^ and *σ*_cyto_ = 0.32 ± 0.05 S/m, whereas the measurement results after stretching were *C*_mem_ = 13.23 ± 0.32 mF/m^2^ and *σ*_cyto_ = 0.35 ± 0.06 S/m. As shown in Fig. 5a, the small difference implies that optical stretching has little effect on the single-cell electrical property measurement. Using numerical methods, we obtained the sensitivity for the unit membrane capacitance (∼0.01 mF/m^2^) and the sensitivity for cytoplasmic conductivity (∼0.002 S/m).

In our other measurement experiments, each cell sample was subjected to in situ stretching and rotation, so its mechanical and electrical properties were collected together to obtain the type-wise mechanical and electrical properties summarized in Tables 1 and 2, respectively. To study the underlying link of both properties, Fig. 5b shows the distribution of the five parameters after normalization for ten HeLa cells. We then plotted the distribution of the five parameters after normalization for all five types of cells, as shown in Fig. 5c. The results reveal the difference between cell types, but the parameters that can reflect the difference most are a different set against *σ*_cyto_ and *τ*. This finding indicates that a certain single parameter of mechanical or electrical properties may fail to specify cell types. By contrast, the combination of those parameters is most likely to better characterize cells. To demonstrate this idea, the type-wise values of the five property parameters were processed by principal component analysis, and the result is shown in Fig. 5d. It can be seen that the first two principal components of the five parameters are able to distinguish the cell type.

### Altered properties of TGF-β1-treated EMT A549 cells

**Fig. 6:**
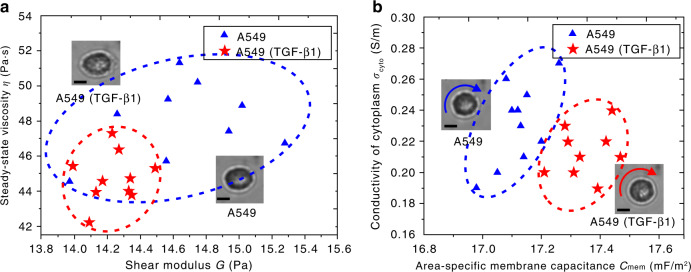
Property measurement results of A549 cells w/o and with TGF-β1 treatment. **a** Mechanical properties. **b** Electrical properties. Insets show the typical morphology of cells under stretch and rotation w/o and with TGF-β1 treatment. Scale bars: 5μm

TGF-β1 has been well established as an EMT inducer in different cell lines such as A549. Our experimental results show that after TGF-β1 treatment, A549 cells changed their mechanical properties by increasing the average deformation from 13.5 to 13.9%. According to the results of optical stretch, we calculated and plotted the average shear modulus and steady-state viscosity of twenty A549 cells without and with TGF-β1 treatment, as shown in Fig. 6a. On average, the shear modulus and steady-state viscosity of A549 cells were 2.7% and 6.6% smaller, respectively, after TGF-β1 treatment.

Through the electrorotation experimental results, we found that the electrical properties of the cells changed after TGF-β1 treatment. Figure 6b shows the area-specific membrane capacitance and cytoplasmic conductivity of twenty A549 cells without and with TGF-β1 treatment. On average, area-specific membrane capacitance was 1.3% greater, while cytoplasmic conductivity was 8.7% less after TGF-β1 treatment. This quantitative difference may provide new thoughts for biologists to investigate cancer cell development and progression.

## Discussion

The cell phenotype can be affected by DEP-related manipulations. In this sense, it is always desirable to conduct cell property measurements in cell medium, which would more closely reflect the cell’s phenotype. However, a cell medium with high conductivity is not suitable for DEP manipulation. For the DEP-related electrical property measurement method to work in this device, DEP buffer with low conductivity had to be used, as opposed to high conductivity solutions prone to problems such as Joule heating and electroosmotic flow.

In addition, the same cell is measured in situ for both mechanical and electrical properties. To this end, it is unnecessary to use different solutions for the two techniques. Therefore, in this work, we used DEP buffer for mechanical property measurement as well. This fact may be one limitation of our method, as DEP buffer may detrimentally affect the mechanical property measurement. On the other hand, now that the electrical property measurement must be in DEP buffer regardless, the necessity of using another solution (e.g., cell medium) for the same cell’s mechanical property measurement turns out to be a minimal issue. Furthermore, according to recent literature^16^, it seems more important to use the same solution for different cells than some specific solution to collect comparable measurement results.

Different media may directly impact the mechanical properties of the cell to be measured. For example, the literature^26,47^ shows that the pH value, temperature, isotonic pressure, and ion concentration of the solutions would affect the morphology and thus mechanical properties of the cells. However, from the measurement side, if we look into the current analytical model as shown by Eq. (5), the refractive index of the medium is the only medium-associated parameter that affects the mechanical measurement^57^. Supposing that the cell maintains its mechanical properties in cell medium (refractive index *n*_medium_ = 1.335)^58^, the mechanical measurement would increase in theory by 1% with respect to the DEP buffer (refractive index *n*_DEP_ = 1.327) used in the experiment. The difference is negligible.

This device is suitable for manipulations such as capture, trapping, stretching, and rotation of both spherical and nonspherical cells. However, the analytical equations for mechanical and electrical property measurement here are derived for spherical and homogeneous cells, so they cannot be directly applied for nonspherical cells. To extend the potential of the device, we plan to establish analytical models for nonspherical cells in future work. In that case, we probably have to consider the geometry and structure of specific cells (e.g., biconcave red blood cells, rod-shaped bacterial cells) to decide how to modify or reconfigure the device for optical trapping and electrorotation of cells.

## Conclusions

We combine the optical stretching and electrorotation modules in one microfluidic chip to facilitate multiple physical parameter measurements for single cells. In particular, two optical fibers are embedded into one of the two orthogonal microchannels formed by four isolated 3D electrodes sitting on a transparent substrate. The two opposing fibers generate optical stretching to efficiently capture trap–stretch cells and maintain the spatial position of the cell for stable rotation in the subsequent electrorotation enabled by the four 3D electrodes. Through optical stretching and electrorotation, we were able to obtain five mechanical and electrical properties of the cell in the same microfluidic chip. We provided proof of concept by testing five types of cells including normal cells (HepaRG and MCF10A) and cancer cells (HeLa, A549, and MCF7), and we demonstrated its potential in cancer-related cell analysis. Considering its easy-to-fabricate and easy-to-operate merits, we believe that this integrated chip can be a powerful tool in single-cell manipulation and analysis.

## Supplementary information

ESI Video 1

ESI Video 2

ESI Video 3

ESI Video 4

ESI Video 5

Figure 1
