# Supplementary figures and images for: On-chip integrated optical stretching and electrorotation enabling single-cell biophysical analysis
Source: Microsyst Nanoeng. 2020 Jun 15;6:57. doi: 10.1038/s41378-020-0162-2 (PMC8433418; doi:10.1038/s41378-020-0162-2)

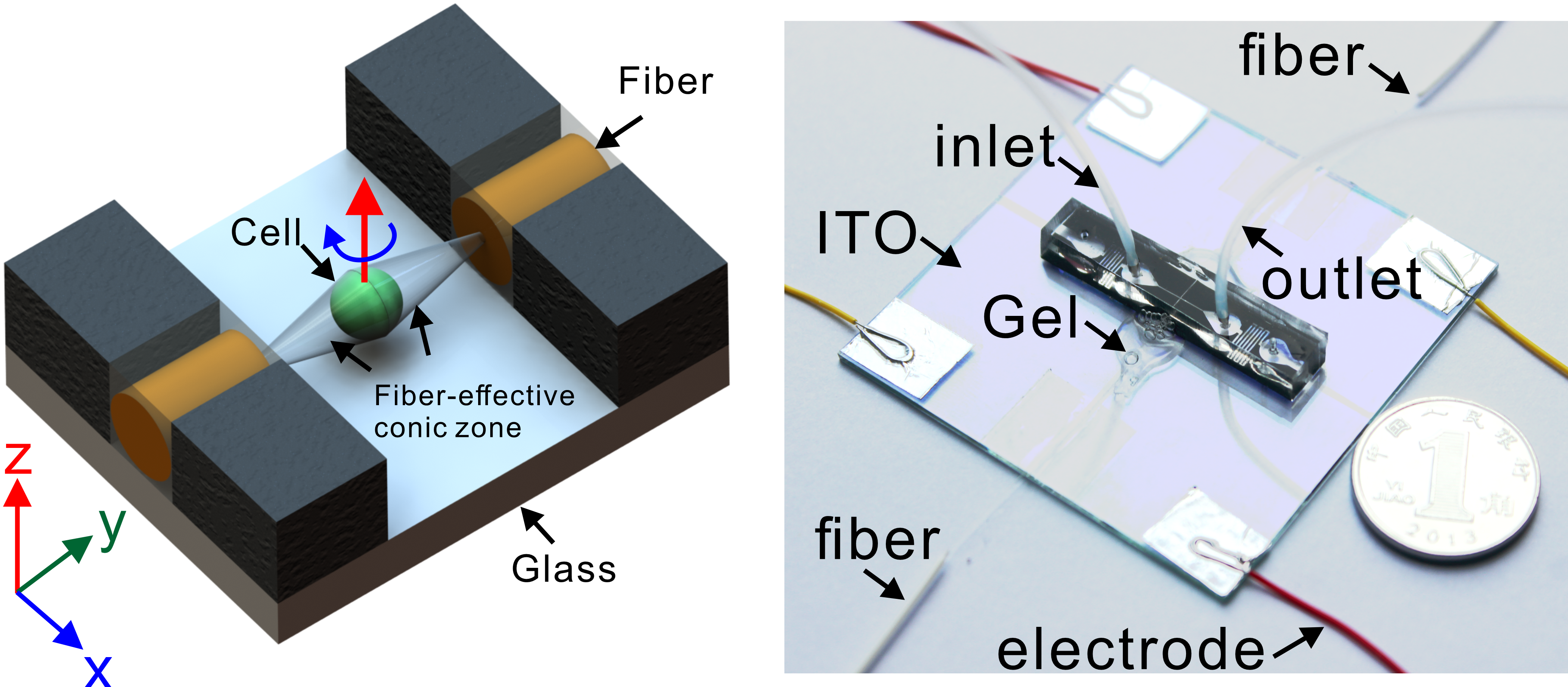

Supplement: Supplementary file 6 — Figure 1 [file 41378_2020_162_MOESM6_ESM.tif]
